# A Novel Prediction Tool for Endoscopic Intervention in Patients with Acute Upper Gastro-Intestinal Bleeding

**DOI:** 10.3390/jcm11195893

**Published:** 2022-10-05

**Authors:** Ido Veisman, Amit Oppenheim, Ronny Maman, Nadav Kofman, Ilan Edri, Lior Dar, Eyal Klang, Sigal Sina, Daniel Gabriely, Idan Levy, Dmitry Beylin, Ortal Beylin, Efrat Shekel, Nir Horesh, Uri Kopylov

**Affiliations:** 1 Department of Gastroenterology, Sheba Medical Center, Tel Hashomer, Ramat Gan 52620, Israel; 2 Faculty of Medicine, Tel-Aviv University, Tel Aviv 67011, Israel; 3 Department of Medicine A, Sheba Medical Center, Tel Hashomer, Ramat Gan 52620, Israel; 4 Department of Surgery and Transplantation, Sheba Medical Center, Tel Hashomer, Ramat Gan 52620, Israel; 5 Department of Surgery, Wolfson Medical Center, Holon 5822012, Israel; 6 Sami Sagol AI Hub, ARC, Sheba Medical Center, Tel Hashomer, Ramat Gan 52620, Israel; 7 Faculty of Medicine, The Hebrew University, Jerusalem 9112102, Israel

**Keywords:** upper GI bleeding, machine learning, Glasgow-Blatchford score (GBS), pre-endoscopic Rockall score

## Abstract

(1) Background: Predicting which patients with upper gastro-intestinal bleeding (UGIB) will receive intervention during urgent endoscopy can allow for better triaging and resource utilization but remains sub-optimal. Using machine learning modelling we aimed to devise an improved endoscopic intervention predicting tool. (2) Methods: A retrospective cohort study of adult patients diagnosed with UGIB between 2012–2018 who underwent esophagogastroduodenoscopy (EGD) during hospitalization. We assessed the correlation between various parameters with endoscopic intervention and examined the prediction performance of the Glasgow-Blatchford score (GBS) and the pre-endoscopic Rockall score for endoscopic intervention. We also trained and tested a new machine learning-based model for the prediction of endoscopic intervention. (3) Results: A total of 883 patients were included. Risk factors for endoscopic intervention included cirrhosis (9.0% vs. 3.8%, *p* = 0.01), syncope at presentation (19.3% vs. 5.4%, *p* < 0.01), early EGD (6.8 h vs. 17.0 h, *p* < 0.01), pre-endoscopic administration of tranexamic acid (TXA) (43.4% vs. 31.0%, *p* < 0.01) and erythromycin (17.2% vs. 5.6%, *p* < 0.01). Higher GBS (11 vs. 9, *p* < 0.01) and pre-endoscopy Rockall score (4.7 vs. 4.1, *p* < 0.01) were significantly associated with endoscopic intervention; however, the predictive performance of the scores was low (AUC of 0.54, and 0.56, respectively). A combined machine learning-developed model demonstrated improved predictive ability (AUC 0.68) using parameters not included in standard GBS. (4) Conclusions: The GBS and pre-endoscopic Rockall score performed poorly in endoscopic intervention prediction. An improved predictive tool has been proposed here. Further studies are needed to examine if predicting this important triaging decision can be further optimized.

## 1. Introduction

Acute upper gastro-intestinal bleeding (UGIB) is a common and urgent medical condition, usually requiring hospital admission with reported annual incidences in the range of 48 to 172 per 100,000 [1]. Current guidelines recommend that following hemodynamic resuscitation, patients with UGIB should undergo an esophagogastroduodenoscopy (EGD) within 12–24 h of presentation [2,3]. Endoscopic therapy in the setting of acute UGIB is indicated when recent bleeding stigmata is observed via EGD, leading to a reduction in the rates of mortality, recurrent bleeding, and surgical intervention [4,5,6]. Several guidelines support utilization of pre-endoscopic assessment scores to stratify patients to low and high-risk groups [2,7]. The Glasgow-Blatchford score (GBS) is a risk assessment tool, synthesizing clinical and laboratory parameters to identify the likelihood of a patient to require medical intervention such as endoscopy, blood transfusion or surgery [8]. The pre-endoscopic Rockall score is an assessment tool which was designed to predict mortality among UGIB patients [9]. In 2017, a prospective study by Stanley and colleagues suggested that only the GBS, and not the other accepted scores (i.e., Rockall and AIM 65), is a reliable score for a composite outcome of transfusion, hemostatic intervention, or death. However, when assessing the performance of the scores for an endoscopic intervention solely, the area under the receiver operating characteristic curve (AUROC) is less than 0.80 for each of the scores, including the GBS [10]. Hence, the clinical utility of the current scores for this crucial outcome is limited. Prediction of endoscopic therapy among acute UGIB patients is complex and has significant implications in patient management. The purpose of this study was to identify parameters in correlation with endoscopic intervention and to create a dedicated predictive model for endoscopic intervention exclusively, in patients with acute UGIB. The new model is based on the GBS and other parameters found significantly associated with endoscopic therapy.

## 2. Materials and Methods

### 2.1. Study Design and Patient Selection

This was a retrospective study including consecutive adult patients who were hospitalized at the Sheba medical center due to acute UGIB, melena or hematemesis between 2012–2018, and had an EGD during their respective hospitalization. Data were collected from an electronic repository including demographic, hemodynamic and laboratory variables at presentation, necessary to calculate GBS and pre-endoscopic Rockall score. Other clinical variables, laboratory results, medical history, free-text physician records and endoscopy reports were collected as well.

### 2.2. Data Extraction

The following data were collected from the electronic health record of the Sheba Medical Center:

- Demographic factors—age in years and sex.
- Comorbidities—including hypertension (HTN), diabetes mellitus (DM), cardio-vascular disease (i.e., ischemic heart disease (IHD), arrythmias, valvular disorder, stroke), pulmonary disease (i.e., asthma, chronic obstructive pulmonary disease (COPD)), deep vein thrombosis (DVT), pulmonary embolism (PE) and cirrhosis.
- Chronic treatment of anticoagulants and anti-platelets—including aspirin, P2Y_12_ inhibitors (clopidogrel, ticagrelor, prasugrel), warfarin and direct oral anticoagulants (DOACs, i.e., dabigatran, rivaroxaban, apixaban).
- Hemodynamic, laboratory variables—all necessary variables at presentation for GBS calculation were collected. Other laboratory results including C-reactive protein (CRP), white blood count (WBC), platelets count, international normalization ratio (INR) and albumin were collected as well.
- Medications—date regarding blood transfusion during hospital stay and treatment with tranexamic acid (TXA, i.e., Hexakapron), proton pump inhibitors (PPI), erythromycin and intravenous (IV) fluids prior to endoscopy were collected.
- Endoscopic data—data regarding endoscopic diagnosis and endoscopic intervention were collected. Endoscopic intervention was defined as the use of at least one or a combination of the following interventions—band ligation, adrenaline injection, hemoclip application and/or coaptive coagulation. All EGD were performed by a trained gastroenterologist or physicians in their GI training under the supervision of a senior gastroenterologist.

### 2.3. Study Aim

The main study goal was to assess the possible correlation between baseline parameters and endoscopic intervention. Furthermore, we assessed the ability of the GBS and the pre-endoscopic Rockall score to predict endoscopic intervention and to create a dedicated model for intervention prediction. The main study outcome was endoscopic intervention as defined in the data extraction section.

### 2.4. Data Analysis

Random forest models were trained to predict the two study outcomes. Data pre-processing included median imputation of missing values. Threefold cross validation splits were employed in each experiment. The testing-folds results were averaged to achieve pooled metrics. Single feature analysis was used to establish the optimal features to be used in the models. The final models’ metrics included area under the receiver operator curve (AUC), sensitivity, specificity, PPV, NPV, and F1. For the explainability of variables in the random forest models, we used feature importance and Shapley additive explanations (SHAP) values. Machine learning programming was carried out with Python (Version 3.7, 64 bits).

### 2.5. Statistical Analysis

Categorical variables were presented as frequency and percentage and as medians and intra-quartile ranges (IQR) for continuous variables. Statistical significance for comparison of continuous variables was evaluated using the Student’s *t*-test/Kruskal–Wallis test and Chi-square test/Fisher’s exact test for categorical variables. Pearson coefficient evaluated linear correlation between the features in the models. All statistical tests were two-sided, and a *p* value < 0.05 was considered statistically significant. Statistical analysis was performed using with Python (Version 3.7 64 bits).

### 2.6. Study Ethics and Patient Consent

This study was carried out in accordance with the ethical guidelines of the Declaration of Helsinki. Since this was a retrospective analysis, no informed consent was obtained.

## 3. Results

This section may be divided by subheadings. It should provide a concise and precise description of the experimental results, their interpretation, as well as the experimental conclusions that can be drawn.

### 3.1. Patient Characteristics

A total of 883 patients were included in this study. A total of 52 (62.5%) were male, the median patient’s age was 69.0 (IQR 58.0–79.0). A total of 579 patients (65.5%) had endoscopic intervention and/or blood transfusion. Endoscopic intervention without blood transfusion was performed in 145 (16.4%) patients and 434 patients were treated with blood transfusion with no endoscopic intervention. The etiology of bleeding for which endoscopic treatment was performed are presented in supplementary Table S1. In-hospital mortality rate was 4.3% (*n* = 38). All patients underwent EGD within 120 h from admission. The median GBS was 9.0 (IQR 6.0–12.0) and median time to endoscopy was 16 h (IQR 5.7–24.03). All demographic and clinical data of endoscopic intervention and in-hospital treatment are depicted in Table 1.

### 3.2. Univariate Analysis of Risk Factors Associated with Endoscopic Intervention

#### 3.2.1. Background Diagnosis and Clinical Features and Endoscopy Timing

Several parameters correlated with increased risk for endoscopic intervention as presented in Table 1. Age, gender, and comorbidities except cirrhosis, did not correlate with endoscopic intervention. Cirrhosis (9.0% vs. 3.8%, *p* = 0.01) and syncope at presentation (19.3% vs. 5.4%, *p* < 0.01) were found to correlate with a higher rate of endoscopic intervention. Higher GBS (11 vs. 9, *p* < 0.01) and higher pre-endoscopic Rockall score (4.7 vs. 4.1, *p* < 0.01) were significantly associated with endoscopic therapy. Patients who underwent EGD earlier were more likely to be treated endoscopically (median 6.8 h [IQR 3.2–16.4] vs. median 17.0 h [IQR 8.6–25.0] *p* < 0.01).

#### 3.2.2. Correlation between Medical Therapies and Endoscopic Intervention

Administration of TXA (43.4% vs. 31.0%, *p* < 0.01) and erythromycin (17.2% vs. 5.6%, *p* < 0.01) during admission, prior to endoscopy, was associated with higher rates of endoscopic intervention. A total of 47/883 patients in this cohort were regularly treated with DOAC’s, among them only 3/145 patients (2.1%) underwent endoscopic intervention, which was numerically lower, although not statistically significant, in comparison to patients treated with DOAC’s who underwent EGD without therapy (*n* = 44/738 (6%), *p* = 0.08). No correlation was seen between chronic treatment with vitamin K antagonists (6.9% vs. 7.9%, *p* = 0.82), enoxaparin (4.8% vs. 4.7% *p* = 0.86) or anti platelets agents—(acetylsalicylic acid [32.4% vs. 30.1%, *p* = 0.64], P2Y12 inhibitors [12.4% vs. 10.4% *p* = 0.57]) and endoscopic intervention. Fluid administration was not significantly different between the intervention and non-intervention groups.

### 3.3. GBS and Pre-Endoscopic Rockall Score Performance for Prediction of Endoscopic Intervention

GBS and pre-endoscopic Rockall score performance in predication of endoscopic intervention was examined. AUC of 0.54 for GBS and 0.56 for pre-endoscopic Rockall score were demonstrated (Table 2). When assessing the scores’ discriminative ability for the composite outcome of endoscopic intervention and packed blood cell transfusion, the GBS was found to be superior to the pre-endoscopic Rockall score (AUC of 0.70 vs. 0.56, respectively, Table 3).

**Figure 1:**
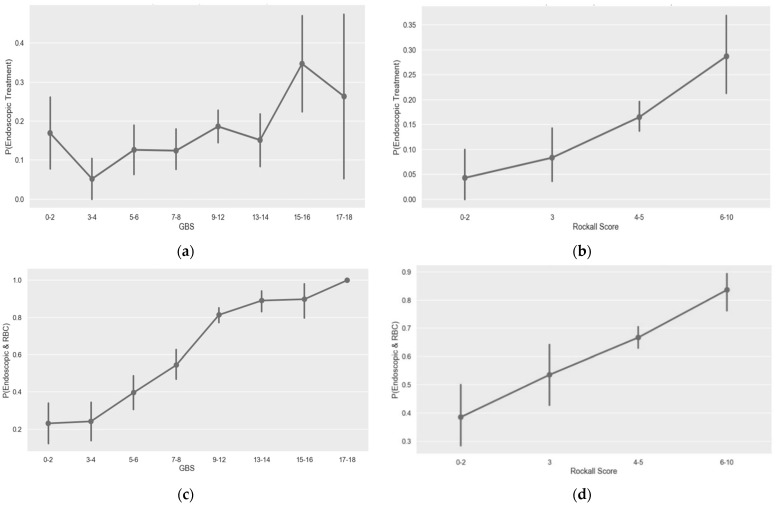
Probability for endoscopic intervention—GBS (**a**) or Rockall score. (**b**) Risk for endoscopic intervention and packed blood cells transfusion—GBS (**c**) or Rockall score. (**d**) *p*, probability; GBS, Glasgow-Blatchford score.

Evaluating the GBS by score groups showed an obvious correlation with the composite outcome of endoscopic intervention and red blood cells (RBCs) transfusion; this correlation was almost completely lost when using the GBS for endoscopic intervention alone. However, the pre-endoscopic Rockall score validity was partially kept when evaluated by score groups for both the composite outcome and endoscopic intervention alone (Table 3, Figure 1a–d).

### 3.4. Predictive Model for Endoscopic Intervention

**Figure 2:**
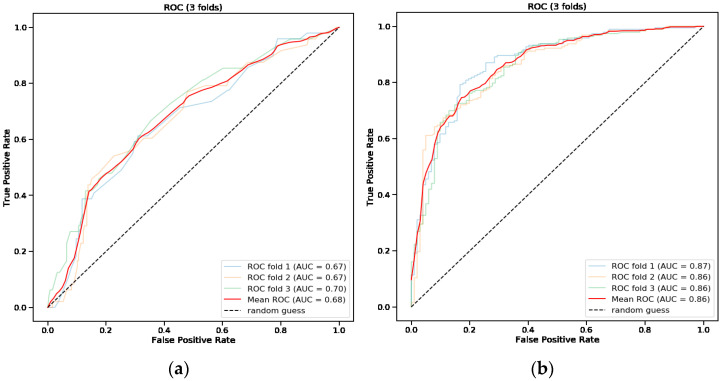
Receiver operating characteristic (ROC) curves * of the new modified model for endoscopic intervention (**a**) and packed blood cells transfusion (**b**). * Cross-validation curves and their mean. AUC, area under the curve.

**Figure 3:**
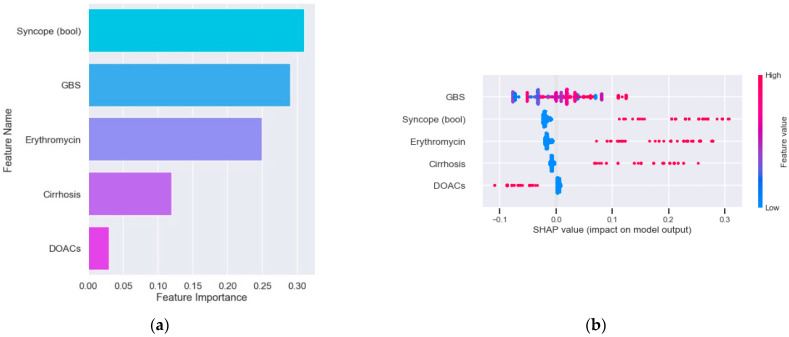
Feature importance in the new modified model for endoscopic intervention. (**a**) SHAP impact plot of the new modified model for endoscopic intervention. (**b**) GBS, Glasgow-Blatchford score; DOACs, direct oral anticoagulants.

The mean AUC of the new model for endoscopic intervention was 0.68 (Table 2, Figure 2a). When assessing the feature importance of all variables included in the model for endoscopic intervention, syncope at presentation, GBS and erythromycin treatment were found to score the most important (Figure 3a). The SHAP importance plot of the model for endoscopic intervention shows variable findings regarding the GBS (i.e., there is no clear correlation between the score and the model prediction) (Figure 3b). The plot does show clear evidence that syncope, cirrhosis and erythromycin use are correlated positively with the risk of intervention and interestingly that DOAC use is negatively correlated with that risk.

### 3.5. Predictive Model for Endoscopic Intervention and Blood Transfusion

A random forest model for the composite outcome of endoscopic intervention and RBCs transfusion was used. Figure 2b shows the receiver operating characteristic (ROC) curves of the validation folds of the model and mean, for the composite outcome. The mean AUC for the composite outcome was 0.86 (Table 3) compared with 0.70 of the GBS. Feature importance analysis of the model for the composite outcome showed hemoglobin level to have the greatest influence on the model with more than threefold the weight of the next feature.

## 4. Discussion

Acute upper GI bleeding still poses a burden on healthcare systems around the world [11,12]. Several scores have been developed to predict clinically relevant outcomes [8,9,13]. However, since these scores were originally published, major changes occurred in the incidence of UGIB, features of patients, management, outcomes [14], and there are different characteristics in UGIB cases worldwide [14,15,16]. Moreover, none of these scores was designed to predict the likelihood of endoscopic intervention exclusively, which is a significant part of the management of UGIB, as previous studies demonstrated correlation between endoscopic intervention and reduced morbidity and mortality [6,17].

The aim of this study was to assess the possible correlation between baseline parameters and endoscopic intervention and to evaluate the performance of the GBS and the pre-endoscopic Rockall score in predicting endoscopic intervention.

The GBS, first published in 2000, is a well-established score used to define patients with UGIB who may be managed safely as outpatients^8^. The score was originally designed to predict a composite outcome including the risk of a blood transfusion, intervention to control bleeding, rebleeding, or death [8]. However, the decision to administer a blood transfusion is based on clinical evaluation and hemoglobin levels. Therefore, the value of predicting this decision is negligible in comparison to the need to predict endoscopic intervention. Testing the predictive performance of the GBS in our study, for endoscopic intervention alone, and for a composite outcome including blood transfusion, we found it to be low.

The pre-endoscopic Rockall is another well-established score, designed for mortality risk assessment only [9]. In our study, like several studies before [10,18], the ability of the pre-endoscopic Rockall score, like the GBS, to predict endoscopic therapy exclusively, is modest.

In a univariate analysis we found the history of cirrhosis, syncope at presentation, pre-endoscopic erythromycin and TXA treatment, and earlier endoscopy time to correlate significantly with endoscopic intervention.

Erythromycin is a macrolide antibiotic with prokinetic activity. In 2016, a meta-analysis by Rahman et al. demonstrated that erythromycin infusion prior to upper endoscopy significantly improved gastric mucosa visualization and reduced the need for a “second-look” endoscopy. However, correlation between erythromycin administration and endoscopic intervention was not assessed [19].

The American College of Gastroenterology recommends the use of erythromycin prior to endoscopy [20]. While acknowledging lack of evidence for erythromycin benefit in reducing further bleeding and mortality, it does provide meaningful reductions in repeat endoscopies and length of hospitalization. Considering its relatively low cost and ease of administration, the panel published a conditional recommendation for its use [20]. On the other hand, the European Society of Gastrointestinal Endoscopy (ESGE) recommends administration of erythromycin only in select patients with severe or ongoing bleeding [2].

In our study, only a minority of patients with UGIB were treated with erythromycin prior to endoscopy, yet a significant correlation was demonstrated between its use and endoscopic therapy. This is probably due to its prokinetic qualities, expelling blood clots distally out of the stomach and proximal duodenum, rendering them clearer to careful visualization and respective endoscopic intervention. On the other hand, its use may be interpreted as a marker for more severe patients, evaluated by the treating physician to have more significant bleeding, and hence treated with erythromycin, to improve the endoscopy outcomes. In both cases, the strong positive correlation herein found reinforces the importance of utilizing this medication among patients with UGIB before endoscopy is performed.

TXA is an antifibrinolytic agent, widely used for several indications, including postpartum hemorrhage, menorrhagia, trauma-associated hemorrhage and surgical bleeding [21]. However, previous studies demonstrated that the efficacy of TXA in patients with upper gastrointestinal bleeding is poor and carries a risk for thromboembolic events [22]. One third of the patients in this study have been treated with TXA and a significant positive correlation between its use and endoscopic intervention was demonstrated. We might hypothesize that bleeding cessation affords better visualization and higher rates of endoscopic intervention. Alternatively, bleeding cessation affords better hemodynamics, for continuation and prolongation of the upper endoscopy, creating a better opportunity to locate and treat a bleeding site. Nevertheless, mortality and other clinical outcomes including thromboembolic events were not assessed in our cohort, hence we cannot recommend its use, based upon our data.

We observed a negative trend between DOAC’s treatment and endoscopic intervention. It can be assumed that although patients receiving this class of anticoagulants tend to bleed, they suffer from bleeding sources that often do not require endoscopic intervention, as has already been reported for dabigatran, which may cause a longitudinal esophageal mucosal injury in approximately 20% of patients [23]. In addition, it can be assumed that due to low availability of an antidote during the study years, a concern to perform endoscopic intervention in these patients led to the avoidance of endoscopic intervention.

The GBS and the pre-endoscopic Rockall score performances in prediction of endoscopic intervention in this tertiary center-based cohort were found to be low. Aiming to build a better prediction tool, random forest models were trained and validated. The performance of the new model was better than the performance of the GBS and the pre-endoscopic Rockall score (AUC of 0.68 vs. 0.54 and 0.56, respectively), yet far from perfect. In evaluating the reasons for the limited performance of the new model, its relative low sensitivity of 0.54 stands out. The specificity of the model was reasonable, much better than the GBS (0.71 vs. 0.28), but certainly not optimal and less than the pre-endoscopic Rockall score with specificity of 0.88. It should be considered that in a predictive model of this kind, where intervention and non-intervention have major risks associated, both the sensitivity and specificity are important for the potential physician using the tool, and so both should be improved in order for this tool to become practical, as should be also represented by a higher AUC.

It should be mentioned that the mortality rate and severe morbidity such as cirrhosis was low in our cohort (4.3% and 4.6% in accordance) compared to data from previous studies worldwide [24,25,26,27]. These differences, indicating different morbidity among different populations may explain the low level of accuracy relative to past studies of the models tested.

Our study, like any study designed to predict endoscopic intervention, has the inherent limitation of an outcome which is subjected to the endoscopist consideration. It is obvious that different physicians may decide differently when encountering similar lesions, based on their training and experience. Their decision may also be affected by the conventions in their units and by the clinical setup, such as timing of procedure, and the assisting nursing team. It should be mentioned that at the Sheba Medical Center, the endoscopist does not receive a fee for endoscopic intervention and hence this factor cannot influence the discretion of the performing endoscopist. This subjective aspect of the outcome limits the generalizability of the study, like any other study in this area. It also limits the potential prediction performance of the model.

## 5. Conclusions

Prediction of endoscopic intervention in UGIB is complex. In this study, we have demonstrated several parameters that significantly correlate with endoscopic intervention. The GBS and pre-endoscopic Rockall score performed poorly in endoscopic intervention prediction, compared with previous studies, which may reflect differences between populations. An improved model has been proposed here; however, its accuracy for prediction of endoscopic intervention was modest. Further research is required to improve this model’s performance, and to examine it in a prospective manner, to make it practical for use in clinical settings. Optional means to improve this model are by expanding the cohort used for the random forest models training, include previous endoscopic evaluation and interventions, include non-invasive imaging raw data using image recognition methods, and possibly incorporating natural language processing tools to analyze free text from the electronic medical record. There is a reasonable chance that using all these methods together will improve the model, and provide a better prediction performance in future versions of it.

## Figures and Tables

**Table 1:** Clinical characteristics of the included patients.

Parameters		Total *N* (%)/Median (IQR) 883 Patients	Endoscopic Intervention *N* (%)/Median (IQR) 145 Patients	No Endoscopic Intervention *N* (%)/Median (IQR) 738 Patients	*p*-Value
Number of patients (%)		883 (100%)	145 (16.4%)	738 (83.6%)	
Age (median, IQR)		69.0 (58.0–79.0)	68.0 (57.0–75.0)	69.0 (59.0–80.0)	0.14
Male gender (*N*, %)		552 (62.5%)	90 (62.1%)	462 (62.6%)	0.97
Time to endoscopy (hours)-(median, IQR)		16.0 (5.7–24.03)	6.8 (3.17–16.37)	17.0 (8.6–24.96)	**<0.01**
Pre endoscopy Rockall Score (mean ± SD)		4.2 ± 1.4	4.7 ± 1.3	4.1 ± 1.4	**<0.01**
GBS (median, IQR)		9.0 (6.0–12.0)	11.0 (8.0–13.0)	9.0 (6.0–12.0)	**<0.01**
Pre- endoscopy treatment (N, %)	Erythromycin	66 (7.5%)	25 (17.2%)	41 (5.6%)	**<0.01**
	TXA	292 (33.1%)	63 (43.4%)	229 (31.0%)	**<0.01**
	Fluids	433 (49.0%)	77 (53.1%)	356 (48.2%)	0.32
	PPI	781 (88.4%)	132 (91.0%)	649 (87.9%)	0.35
	PCC	15 (1.7%)	4 (2.8%)	11 (1.5%)	0.46
	Vitamin K	90 (10.2%)	17 (11.7%)	73 (9.9%)	0.60
	VKA	68 (7.7%)	10 (6.9%)	58 (7.9%)	0.82
Chronic treatment (N, %)	DOACs	47 (5.3%)	3 (2.1%)	44 (6.0%)	0.08
	P2Y12 inhibitors	95 (10.8%)	18 (12.4%)	77 (10.4%)	0.57
	Acetylsalicylic acid	269 (30.5%)	47 (32.4%)	222 (30.1%)	0.64
	Enoxaparin	42 (4.8%)	7 (4.8%)	35 (4.7%)	0.86
INR (median, IQR)		1.09 (0.98–1.27)	1.13 (0.99–1.32)	1.08 (0.98–1.26)	0.12
HGB (median, IQR)		9.25 (7.5–11.2)	8.89 (7.31–10.85)	9.34 (7.5–11.27)	0.22
Heart rate (median, IQR)		89.0 (76.0–100.0)	92.0 (77.0–102.0)	88.0 (76.0–100.0)	0.07
MAP (median, IQR)		86.33 (75.67–95.33)	84.67 (74.0–95.33)	87.0 (76.33–95.58)	0.17
Syncope (*N*, %)		68 (7.7%)	28 (19.3%)	40 (5.4%)	**<0.01**
Cirrhosis (*N*, %)		41 (4.6%)	13 (9.0%)	28 (3.8%)	**0.01**
Cardiac arrhythmia (*N*, %)		84 (9.5%)	18 (12.4%)	66 (8.9%)	0.25
CHF (*N*, %)		95 (10.8%)	18 (12.4%)	77 (10.4%)	0.57
IHD (*N*, %)		144 (16.3%)	21 (14.5%)	123 (16.7%)	0.59
Renal failure (*N*, %)		56 (6.3%)	11 (7.6%)	45 (6.1%)	0.62
COPD (*N*, %)		26 (2.9%)	3 (2.1%)	23 (3.1%)	0.67
HTN (*N*, %)		307 (34.8%)	53 (36.6%)	254 (34.4%)	0.69
DM (*N*, %)		202 (22.9%)	31 (21.4%)	171 (23.2%)	0.71
Cardiac valvular disease (*N*, %)		55 (6.2%)	9 (6.2%)	46 (6.2%)	0.86
Asthma (*N*, %)		26 (2.9%)	5 (3.4%)	21 (2.8%)	0.90
Melena (bool) (*N*, %)		556 (63.0%)	92 (63.4%)	464 (62.9%)	0.97
DVT (*N*, %)		15 (1.7%)	2 (1.4%)	13 (1.8%)	0.97
Stroke (*N*, %)		15 (1.7%)	2 (1.4%)	13 (1.8%)	0.97

IQR, intra-quartile range; SD, standard deviation; GBS, Glasgow-Blatchford score; DOACs, direct oral anti coagulants; INR, international normalized ratio; MAP, mean arterial pressure; HGB, hemoglobin; PPI, proton pump inhibitor; PCC, prothrombin complex concentrate; CHF, congestive heart failure; IHD, ischemic heart disease; COPD, chronic obstructive pulmonary disease; HTN, hypertension; DM, diabetes mellitus; VKA, vitamin k antagonist; DVT, deep vein thrombosis.*p*-Values ≤ 0.01 are marked in bold.

**Table 2:** Prediction model performance for endoscopic intervention.

	Glasgow-Blatchford Score	Pre-Endoscopic Rockall Score	New Modified Model *
AUC	0.54	0.56	0.68
TPR (sensitivity)	0.81	0.24	0.55
TNR (specificity)	0.28	0.88	0.71
PPV	0.18	0.29	0.27
NPV	0.88	0.86	0.89

* mean of 3-fold cross-validation split of the new model. AUC, area under the (receiver operator characteristic) curve; TPR, true positive rate; TNR, true negative rate; PPV, positive predictive value; NPV, negative predictive value.

**Table 3:** Prediction model performance for endoscopic intervention and/or packed blood cells blood transfusion.

	Glasgow-Blatchford Score	Pre-Endoscopic Rockall Score	New Modified Model *
AUC	0.70	0.56	0.86
TPR (sensitivity)	0.87	0.18	0.77
TNR (specificity)	0.53	0.93	0.79
PPV	0.78	0.84	0.88
NPV	0.69	0.37	0.65

* mean of 3-fold cross-validation split of the new model. AUC, area under the (receiver operator characteristic) curve; TPR, true positive rate; TNR, true negative rate; PPV, positive predictive value; NPV, negative predictive value.

## Data Availability

Not applicable.

## Supplementary Materials

The following supporting information can be downloaded at: https://www.mdpi.com/article/10.3390/jcm11195893/s1, Table S1: Bleeding etiology among patients that underwent endoscopic therapy.
