# Supplementary material for: A Novel Prediction Tool for Endoscopic Intervention in Patients with Acute Upper Gastro-Intestinal Bleeding
Source: J Clin Med. 2022 Oct 5;11(19):5893. doi: 10.3390/jcm11195893 (PMC9573673; doi:10.3390/jcm11195893)
Supplement: Supplementary file 1 [file jcm-11-05893-s001.zip › jcm-1901436-supplementary.pdf]

Journal of
Clinical Medicine

Table S1. Bleeding etiology among patients that underwent endoscopic therapy.

Etiology of bleeding	Total<br>*N*
Esophageal Varices	11
Esophageal ulcer	6
Mallory-Weiss Tear	16
Gastric Ulcer	36
Gastric Erosions	10
Gastric varices	5
Gastric tumor	5
Gastric Polyp	4
Gastric vascular malformation	8
Duodenal Ulcer	41
Duodenal erosions	2
Vascular Lesion - 2nd Part of Duodenum	1
